# CRC Therapy Identifies Indian Hedgehog Signaling in Mouse Endometrial Epithelial Cells and Inhibition of Ihh-KLF9 as a Novel Strategy for Treating IUA

**DOI:** 10.3390/cells11244053

**Published:** 2022-12-15

**Authors:** Xinhao Zhou, Yiyi Kang, Yuntzu Chang, Siyu Xia, Ming Wu, Jun Liu, Dirong Dong, Wei Zhang, Hong Chen, Hui Li

**Affiliations:** 1 Taikang Medical School (School of Basic Medical Sciences), Wuhan University, Wuhan 430071, China; 2 Clinical Laboratory, Wuhan Fourth Hospital, Wuhan 430030, China; 3 Department of Obstetrics and Gynecology, Zhongnan Hospital of Wuhan University, Wuhan 430071, China

**Keywords:** conditional reprogrammed cells (CRCs), Indian hedgehog (Ihh), intrauterine adhesion (IUA), mouse model, cell therapy

## Abstract

Intrauterine adhesion (IUA) causes menstrual disturbance and infertility. There is no effective treatment available for moderate to severe IUA cases. Stem cell-based therapy has been investigated for treating IUA but is limited in clinical applications due to issues including the precise induction of differentiation, tumorigenesis, and unclear molecular mechanisms. In our recent study, we isolated and expanded the long-term cultures of conditional reprogrammed (CR) mouse endometrial epithelial cells. Treating IUA mice with these CR cells (CRCs) restored the morphology and structure of the endometrium and significantly improved the pregnancy rate. In this study, our data with high-throughput sequencing, CRISPR knockout Ihh^−/−^CRCs, and transplantation identified for the first time that the Indian hedgehog (Ihh) gene plays a critical role in the regulation of endometrial epithelial cell proliferation. We also found that aberrant activated Ihh-krüppel-like factor 9 (KLF9) signaling contributes to the inhibition of normal progesterone receptor (PR) function in IUA mice. Thus, we hypothesized that inhibition of the Ihh-KLF9 pathway may be a novel strategy to treat IUA. Our data demonstrated that treatment with the hedgehog signaling inhibitor Vismodegib restored the morphology, structure, and microenvironment of the endometrium, and greatly improved the pregnancy rate in IUA mice. This study suggests a promising application of hedgehog inhibitors as a targeted drug in the IUA clinic.

## 1. Introduction

Uterine endometrium is a dynamic tissue that is tightly regulated by hormones such as estrogen and progesterone and requires mutual interactions between epithelial and stromal cells [1,2,3]. The uterine endometrial homeostasis is essential for embryo implantation and pregnancy maintenance [4]. Intrauterine adhesion (IUA) occurs when the endometrium is injured or infected and incapable of self-repair [5]. Pregnancy-related curettage or hysteroscopic adhesiolysis causes about 90% of IUA cases [6,7]. The most typical feature of IUA is loss of endometrial epithelium and fibrosis formation. The clinical symptoms of IUA include hypomenorrhea or amenorrhea, pelvic pain, infertility, and repeated miscarriage [7]. Hysteroscopic adhesiolysis is the major clinical treatment for IUA. Other alternative treatments include physical barriers and hormonal therapy [5]. However, these alternative treatments do not have solid efficacy data from large-scale randomized clinical trials [8]. In addition, a high recurrence rate is an unsolved problem for moderate to severe IUA cases [9,10]. Therefore, an effective treatment or drug for IUA therapy is still urgently needed.

During the human menstrual cycle, the uterine endometrium undergoes extensive remodeling, including periodical shedding and the subsequent quick self-renewal without scarring or loss of function. The re-epithelialization of the endometrial surface after the beginning of menstruation (usually within 48 h) is critical for preventing fibrosis formation [11,12]. The specific sub-population of endometrial epithelial cells with stemness has the potential to regenerate and reconstruct the endometrium [13]. However, direct utilization of these endometrial epithelial cells for IUA therapy has been shown to be impractical because of their extremely limited proliferation capacity [12]. In our most recent report, we established the rapid and stable cultures of mouse endometrial epithelial cells (referred to as CRCs in the rest of the text) by the conditional reprogramming (CR) approach [14]. Treating IUA mice with physiological CRCs restores the morphology and structure of the endometrium and significantly improves the pregnancy rate [14]. However, the mechanism underlying the beneficial effects of transplantation of CRCs in IUA mice remains to be elucidated. Moreover, the pathogenesis of IUA is controversial. Several hypotheses have been proposed including fibrosis hyperplasia, abnormal differentiation of stem cells, changes to the uterine microenvironment, and inflammatory response [15]. We expect that CRCs could serve as a physiological model to investigate the mechanisms of IUA and develop a new targeted drug for IUA therapy.

In this study, we carried out transcriptome analysis (RNA-seq) and identified for the first time that the Indian hedgehog (Ihh) gene plays a critical role in the regulation of endometrial epithelial cell proliferation during CRCs transplantation into IUA mice. Injury-induced activation of Ihh signaling up-regulated the expression of krüppel-like factor 9 (KLF9), led to inhibition of luminal progesterone receptor (PR), and disturbed the proliferation of endometrial epithelial cells in IUA mice. Unexpectedly, we found that pretreatment of the Hedgehog pathway inhibitor (Vismodegib) restored the normal microenvironment, morphology, and structure of endometrium and improved the pregnancy rate in IUA mice, showing a promising prospect of Vismodegib for IUA therapy.

## 2. Materials and Methods

### 2.1. Cell Culture

CRCs, mouse endometrial epithelial cells were established and cultured by the CR approach as previously described [14,16]. Briefly, CRCs were co-cultured with irradiated mouse 3T3 cells (YongTech, Shenzhen, China) in a primary epithelial culture basic medium (PECBM) supplemented with 5 to 10 mol/L Y-27632 (Enzo Life Sciences, Farmingdale, NY, USA). HEK293T cells were obtained from ATCC and cultured in DMEM containing 10% FBS. Cells were cultured in a humidified incubator at 37 °C with 5% CO_2_.

### 2.2. Mouse IUA Model and Cells Transplantation

Eight-week-old C57BL female mice were obtained from the Center for Animal Experiment of Wuhan University. All animal experiments were conducted in accordance with the Chinese National Standard Laboratory Animal Guidelines and approved by the Animal Ethics Committee of Wuhan University Centerfor Animal Experiment. The mouse IUA model was established by mechanical damage as previously described [14]. Briefly, the abdominal wall of anesthetized mice was cut longitudinally to expose the uterus. A 27-gauge needle was inserted into the uterine cavity, rotated, and withdrawn 10 times to cause mechanical damage. Mice were randomly divided into three or four groups with three mice in each group: the sham-operated group, injury group, WT CRCs-transplanted group, and plus Ihh^−/−^CRCs-transplanted group. For cells-transplantation, 50 μL of WT or Ihh^−/−^CRCs (1 × 10^6^) suspension was injected into the uterine cavity immediately after the uterine injury. The injury group received 50 μL of PBS. Mice were sacrificed on day 7 of injury/transplantation. The tissue was collected for morphology, RNA, and RNA sequencing analysis.

### 2.3. Masson’s Trichrome (MT) Staining 

Uterine tissue samples were collected and fixed with 4% paraformaldehyde (PFA) before dehydration and paraffin embedding. Then, the paraffin-embedded tissues were sliced into 5μm-thick sections. Sections were stained with Masson’s trichrome (MT) staining kit (Maixin biotech company, Fuzhou, China) to observe the morphology of tissues. 

### 2.4. RNA Extraction, qRT-PCR, and RNA Sequencing Analysis

RNA was extracted from uterine tissues or cells with the RNeasy Mini Kit (74104, Qiagen) according to the manufacturer’sinstructions. HiScript III All-in-one RT SuperMix Perfect for qPCR (R333-01, Vazyme, Nanjing, China) was used for the reverse transcription reaction to obtain cDNA. The cDNA was amplified with the specific primers listed in Table 1. Gene expression analysis was conducted by qRT-PCR with Taq Pro Universal SYBR qPCR Master Mix (Q712-02, Vazyme, Nanjing, China) on a Bio-Rad CFX 96. For RNA sequencing analysis, samples with RNA integrity number (RIN) ≥ 7.5 were subjected to RNA-seq (GEO database GSE217365). RNA libraries were prepared using the Ribo-Zero GoldrRNA Removal (Illumina, San Diego, CA, USA) followed by sequencing on the NovaSeq6000 (Illumina, San Diego, CA, USA). The index-trimmed pair-end 150 base pair reads were aligned to the murine reference genome (mm38) using Hisat2 (v2.1.0) [17,18]. StringTie (v2.1.1) was used to assemble quantitative full-length transcripts representing multiple splice variants for each gene locus [19]. The differentially expressed genes obtained were used to generate heatmaps by applying the R package pheatmap (v1.0.8). Principal component analysis (PCA) was performed using the custom scripts with unsupervised transformed counts. Gene ontology and gene regulatory network analysis was performed using the R Bioconductor cluster Profiler package (v3.14.3) [20].

### 2.5. Generation of Ihh CRISPR/Cas9 and mCherry Lenti-Viral Particles

Ihh-specific guide RNAs (gRNAs) and the Cas9 co-expression lenti-viral vector (dual gRNAs) were obtained from VectorBuilder (Guangzhou, China). This one vector system expresses the gRNAs, Cas9 protein, and puromycin-resistant gene. Two Ihh-specific gRNAs sequences were designed by VectorBuilder: 5′-CTTGCCTTCGTAGCGCCCGC-3′ and 5′-TTTACACTATGAGGGCCGCG-3′ (Figure S1). Ihh CRISPR/Cas9 lenti-virus packaging particles (>10^8^ TU/mL) and mCherry control lenti-virus particles (>10^8^ TU/mL) were generated by VectorBuilder and aliquoted and stored at −80 °C.

### 2.6. Viral Infection and Selection of Ihh^−/−^CRCs

CRCs cells were seeded in a 24-well plate and infected with Ihh CRISPR/Cas9 lenti-virus and mCherry control lenti-virus (viruses were diluted with medium) according to the manufacturer’s protocols. After 24 h post-infection, puromycin (2 μg/mL final concentration) was added to the medium. The subclones of virus-transduced parent CRCs were selected by limited dilutions and expanded. The DNA of subclones was extracted and PCR primers were designed to specifically amplify the chromosome 1 genome regions, 74990079–74990481 (403bp) and 74987501-74988023 (523bp), that cross the Ihh CRISPR-Cas9 cutting sites recognized by gRNA1 and gRNA2, respectively. PCR amplification and DNA agarose gel electrophoresis were performed to obtain the target bands followed by Sanger sequencing.

### 2.7. Immunohistochemical (IHC) and Immunofluorescent (IF) Analysis

The paraffin-embedded sections were dewaxed, rehydrated, and followed by standard IHC protocol [14]. Sections were incubated with primary antibodies (Table 2) at 4 °C overnight, followed by incubation with HRP-conjugated secondary antibodies for 30 min. Immunoreactivity was developed using a DAB substrate kit (Maixin biotech company, Fuzhou, China). Nuclei were counterstained with hematoxylin. Immunofluorescent staining was performed using a similar protocol, followed by incubation with Cy3 or DyLight 488-conjugated secondary antibodies. All the slides were mounted with an anti-fade mount and visualized with an Olympus CKX53 fluorescence microscope (Tokyo, Japan). 

### 2.8. Cell Transfection

pHA-Gli1 and empty vectors were obtained from VectorBuilder (Guangzhou, China). The siRNAs targeting KLF9 were purchased from Sangon Biotechnology (Shanghai, China). CRC transfection was conducted using Lipofectamine 2000 (Thermo Fisher, Waltham, MA, USA) according to the manufacturer’s instructions. Total RNA and cell lysate were harvested at the indicated time points after transfection for further experiments.

### 2.9. Western Blotting Assay

The cell lysate was harvested from cells and standard Western blotting protocols were performed as previously described [21]. The protein samples were specifically probed with primary antibodies (as listed in Table 2). The immunoreactivity signal was visualized using the Omni-ECL™Pico Light Chemiluminescence Kit (SQ202L, Epizyme, Shanghai, China).

### 2.10. Luciferase Reporter Assay

KLF9 firefly luciferase reporter pKLF9-Luc and pRL-TK, plasmid pHA-Gli1, and empty vectors were obtained from VectorBuilder (Guangzhou, China). HEK293T cells were seeded in 24-well plates and co-transfected with pKLF9-Luc and pRL-TK using Lipofectamine 2000 (Thermo Fisher) according to the manufacturer’s instructions. pHA-Gli1 or empty vectors were also co-transfected into HEK293 cells. After 36 h, dual-luciferase activity was determined using the Dual Luciferase Reporter Assay Kit (DL101-01, Vazyme). KLF9 firefly luciferase activity was normalized to Renilla luciferase activity. The results were shown as the means ± SD of triplicate wells. Each experiment was repeated three times.

### 2.11. IUA Mice Treated with Vismodegib

Nine 8-week-old C57BL females were randomly divided into three groups (three mice in each group): the sham-operated group, the injury group, and Vismodegib-treated (injury + Vismodegib) group. Vismodegib was purchased from Selleck Chemicals (Shanghai, China). The dosage of Vismodegib was determined as 90 mg/kg in the mouse model according to a previous study [22]. Six hours before mechanical damage to the uterine endometrium, a single oral gavage of Vismodegib (90 mg/kg) was administrated to the mice of the Vismodegib-treated group. The other two groups were given an equal volume of carrier solution by gavage. All mice were sacrificed on day 7 after surgery and uterine tissues were collected for mRNA expression and morphology analysis. 

### 2.12. Breeding Study with VismodegibTreatment

Another batch of 21 female mice (8-week-old C57BL/6) was randomly divided into three groups (*n* = 14 uterine horns/group): sham-operated group, injury group, and Vismodegib-treated (injury + Vismodegib) group. The intrauterine mechanic damage and Vismodegib gavage were performed as above procedures. Three estrous cycles (14 days) after injury, female mice were bred with 8-week-old C57BL/6 male mice. The day when the vaginal plug was observed was designated as gestation day (GD) 0. After GD 0, male mice were removed to maintain one pregnancy. All female mice were sacrificed for embryo implantation examination at GD 18 with an overdose of sodium pentobarbital (200 mg/kg intraperitoneally).

### 2.13. Statistical Analysis

Data were analyzed using GraphPad Prism 8.0 and presented as the mean ± SD. A two-tailed t test was performed to compare the statistical differences between the two groups, and a one-way analysis of variance (ANOVA) was used to compare differences among multiple groups. Statistical significance was accepted when the *p*-value was <0.05. 

## 3. Results

### 3.1. CRC Transplantation Promotes the Endometrial Restoration in IUA Mice

**Figure 1:**
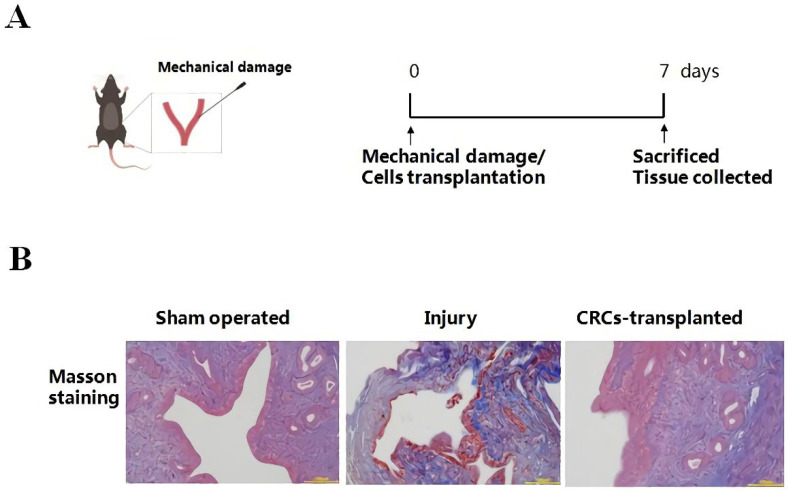
Transplantation of CRCs repairs the injured uterine endometrium in IUA mice. (**A**) A schematic diagram describing the procedure of the IUA mice model and cell transplantation. The IUA mice were established through mechanical trauma to the murine endometrium as described in the Materials and Methods. Cell transplantation to the uterine cavity was carried out immediately after injury (*n* = 3 in each group). (**B**) Masson’s trichrome (MT) staining of the endometrium from three experimental groups of mice. The endometrial tissues were collected after 7 days of injury/cell transplantation. Then tissues were processed by standard histological procedures and stained by Masson’s trichrome to evaluate the degree of endometrial fibrosis. Blue staining indicated fibrosis. Scale bar, 100 μm.

The mouse IUA model was constructed through mechanical damage to the uterine endometrium as previously described [6,14], which recapitulates the morphological features of clinical IUA [23]. The uterine tissues of three experimental groups were collected after 7 days (Figure 1A). Masson’s trichrome staining was carried out to evaluate the structure of the uterine endometrium and the formation of fibrosis in IUA. As shown in Figure 1B, the uterine endometrium of sham-operated control mice was kept intact without fibrosis. In injured mice, the endometrium was disrupted with severe fibrosis (blue staining) and a dramatic reduction in endometrial epithelial cells and glands (Figure 1B). In cells-transplanted mice, the morphology of the endometrium was recovered and fibrosis was obviously ameliorated by CRCs transplantation compared to injured mice (Figure 1B). These results demonstrated that transplantation of CRCs significantly promotes endometrial restoration and inhibits fibrosis in IUA mice, as we reported previously [14].

### 3.2. Transcriptome Analysis Reveals Hub Genes and Ihh and KLF9 Axis in the Endometrial Restoration in IUA Mice

**Figure 2:**
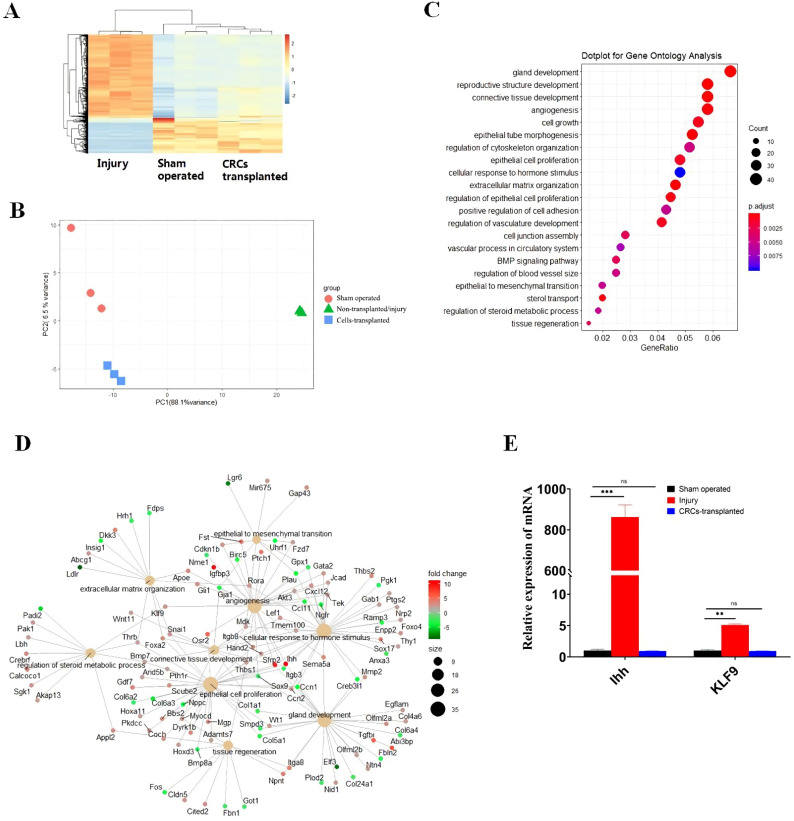
Transcriptomeanalysis reveals hub genes and Ihh and KLF9 axis in the repair of uterine endometrium by CRCs transplantation. (**A**) Differentially expressed gene patterns in three experimental groups of mice. Hierarchical clustering analysis based on genecode (version 23) gene annotation shows that the cells-transplanted group cluster is more closely related to the sham-operated control group. (**B**) Principal component analysis (PCA) in three experimental groups of mice. PCA showed a general clustering of the control group and cells-transplanted group, but more variation with the non-transplanted/injury group. (**C**) Gene ontology (GO) pathway analysis in three experimental groups of mice. GO analysis of differentially expressed genes (DEGs) in the injury group revealed various biological processes. (**D**) Gene regulatory network analysis in three experimental groups of mice. The gene regulatory network analysis of differentially expressed genes (DEGs) revealed several hub genes including Hand2, Ihh, Thbs1, KLF9, Osr2, and Sfrp2. (**E**) Validation of the RNAseq results by RT-qPCR. Total RNA was extracted from the endometrial tissues of three experimental groups of mice. qRT-PCR was carried out to analyze the expression of Ihh and KLF9. The statistical analysis and plotting of the data were completed using GraphPad Prism 8.0. ** indicates *p* < 0.01, *** indicates *p* < 0.001, ns indicates no statistical significance.

To investigate the molecular mechanism during the restoration of mouse endometrium by CRCs transplantation, uterine horns were collected from the mice of the CRC cells-transplanted, injury, and sham-operated groups 7 days after surgery for RNA-sequencing (RNA-seq) [24,25]. Hierarchical clustering analysis and principal component analysis (PCA) showed that the cells-transplanted group clustered more closely to the control group than the injury group (Figure 2A). The results indicated that gene expression shares the same pattern in cells-transplanted and sham-operated mice, while the expression pattern differs from that of the injury mice group (Figure 2B). Gene ontology (GO) analysis of differentially expressed genes (DEGs) in the injury group revealed altered pathways in processes such as gland development, regulation of steroid metabolic process, epithelial cell proliferation, connective tissue development, and cellular response to hormone stimulus(Figure 2C). The gene regulatory network analysis of differentially expressed genes (DEGs) identified several hub genes, including Indian hedgehog (Ihh) and Krüppel-like factor 9 (KLF9), which participated in the restoration of the endometrium by CRCs in IUA mice (Figure 2D). Quantitative real-time PCR (qRT-PCR) validated the up-regulation of these two genes in injured mice (Figure 2E). Ihh is a member of the Hedgehog (Hh) family and is important for the development of multiple tissues [26]. KLF9, also known as basic transcription element-binding protein-1 (BTEB1), is an important member of the KLF/Sp1 family with diverse functions involved in cell proliferation, differentiation, and apoptosis [27]. These results suggested that Ihh and KLF9 may play a critical role during the restoration of endometrium in IUA mice.

### 3.3. Unexpected and Different Roles of Ihh Signaling in CRC Proliferation In Vitro and IUA Repair in Mice

To further investigate the role of Ihh during endometrium restoration and fibrosis, we constructed Ihh knockout cells (Ihh^−/−^CRCs) with the CRISPR/Cas9 approach [28]. The CRISPR/Cas9 lenti-viral vector and gRNAs were designed as shown in Figure S1A,B. The virus-transduced CRCs were cultured in a puromycin medium and subclones were isolated and sequenced (Figure S1C). The knockout of the Ihh gene (clone 3) was confirmed by a Western blotting assay (Figure S1D). The Ihh knockout significantly decreased the growth of cells compared to wild-type (WT) CRCs (Figure S1E). Previous reports have shown that the absence of Ihh in the mouse uterus results in decreased cell cycle progression [29]. Ihh is a regulator of progesterone signaling in the mouse uterus, mediating the communication between the epithelium and stroma and suggesting the feedback regulation between Ihh and PR [4,30]. Our results (Figure S1E) also indicated that Ihh is involved in the regeneration of the endometrium.

**Figure 3:**
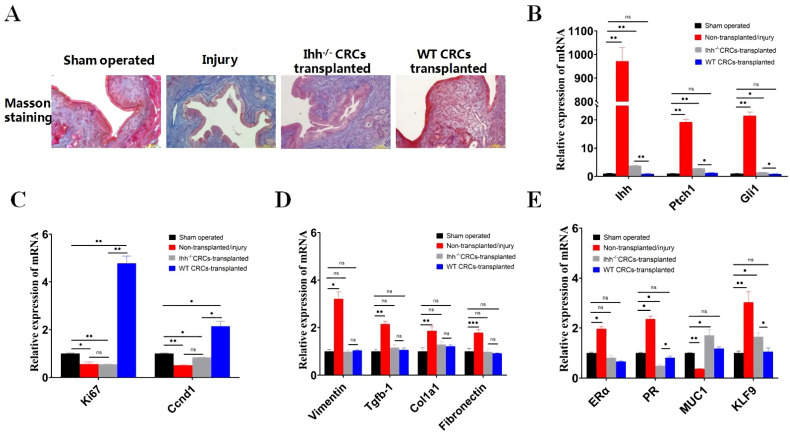
Unexpected and different roles of Ihh signaling in CRC proliferation in vitro and IUA repair in mice. (**A**) The endometrial morphology of the experimental groups of mice after transplantation of Ihh^−/−^CRCs or WT CRCs. Masson’s trichrome (MT) staining was performed to assess endometrial fibrosis. Scale bar, 100 μm. (**B**–**E**) RNA expression of Ihh signaling pathway molecules (**B**), cell proliferation markers (**C**), fibrosis markers (**D**), and key endometrial functional molecules (**E**) in the experimental groups of mice. Uterine endometrial tissues were collected and qRT-PCR analysis was carried out. β-actin was the internal control, and the normalized expression level is displayed in a histogram (* *p* < 0.05, ** *p* < 0.01, *** *p* < 0.001, ns indicates no statistical significance.). The statistical analysis and plotting of the data were completed using GraphPad Prism 8.0.

Next, we investigated the role of the Ihh signaling pathway in uterine endometrial injury and repair by transplantation of wild-type (WT) CRCs and Ihh^−/−^CRCs in IUA mice. Consistent with the results in Figure 1B, Masson’s trichrome staining confirmed the restoration of the endometrium and ameliorated fibrosis by WT CRCs transplantation in IUA mice (Figure 3A). Although fibrosis (blue staining) was reduced and the morphology of the endometrium was recovered in Ihh^−/−^ cells-transplanted mice, the recovery level was less than that in WT cells-transplanted mice (Figure 3A). qRT-PCR results showed that mRNA expression of Ihh and Ihh signaling patched receptor (Ptch) 1 and the glioma-associated oncogene homolog (GLI) 1 was up-regulated in the injury mice group (Figure 3B). Transplantation of WT CRCs significantly down-regulated the expression of Ihh, Ptch1, and Gli1 to a level close to that in control mice (no statistical significance). Transplantation of Ihh^−/−^CRCs also down-regulated the expression of Ihh, Ptch1, and Gli1, but the expression of these genes was higher than those in control and WT cells-transplanted mice (** *p* < 0.01, * *p* < 0.05). 

The proliferative capacity of mouse endometrium in experimental mice was analyzed by the proliferation markers Ki67 and Ccnd1 (Cyclin D1) (Figure 3C). The results showed that the mRNA expression of Ki67 and Ccnd1 in the WT CRCs-transplanted mice was much higher than those in sham-operated mice and injury group mice, indicating the proliferation of CRCs in the luminal endometrium. The mRNA level of Ki67 and Ccnd1 in Ihh^−/−^CRCs transplanted mice was much lower than that in WT cells-transplanted mice (** *p* < 0.01, * *p* < 0.05), which was close to that in injury group mice (no statistical significance). These results demonstrated that transplantation of WT CRCs greatly increased the proliferation of CRCs in the luminal endometrium, while Ihh^−/−^CRCs lost those functions in IUA mice. 

As shown in Figure 3A, obvious fibrosis (blue staining) was observed in injury group mice. The expression of fibrosis markers Vimentin, Tgfb-1, Col1a1, and Fibronect was up-regulated in injured mice compared to that in sham-operated mice (Figure 3D). Transplantation of WT and Ihh^−/−^CRCs both decreased the expression of these fibrosis markers to the level of the sham-operated group. The expression of fibrosis-related markers in the two cells-transplanted groups showed no significant difference.

We further detected the expression of estrogen receptor α (ERα), PR, Mucin 1 (MUC1, a marker of luminal/gland endometrial epithelium), and KLF9 in the uterus of experimental mice (Figure 3E). The results showed that the expression of ERα and PR was up-regulated in injury group mice compared to that in sham-operated mice. Transplantation of WT and Ihh^−/−^CRCs cells both decreased the expression of ERα and PR close to the control level (sham-operated group) while the mRNA level of PR in Ihh^−/−^CRCs cells-transplanted mice was lower than that in WT cells-transplanted mice (Figure 3E).

The expression of MUC1 in the injury group was down-regulated compared to that in the sham-operated group, indicating the loss of endometrial epithelium (Figure 3E). WT and Ihh^−/−^CRCs-transplantation increased the mRNA expression of MUC1 back to the control level. The expression of KLF9 mRNA was significantly up-regulated in the injury group compared to the sham-operated control group (Figure 3E). WT CRCs transplantation reduced the KLF9 expression to the control level. In contrast, Ihh^−/−^CRCs transplantation could only down-regulate KLF9 expression to a certain level which was higher than that of WT cells-transplanted mice (Figure 3E). 

Based on our results (Figure 1) and hypothesis, the re-epithelialization of the endometrial surface after injury (at the very beginning) is critical for preventing fibrosis and endometrium restoration. Although cell proliferation, Ihh signaling in CRC cells (in vitro proliferation phase), and injured tissue (in vivo) are all different stages of the injury, cell proliferation, and in vivo endometrium restoration, our results demonstrated that there were differences in Ihh/Ihh^−/−^CRCs in vivo repair in IUA mice. 

To further validate the results of the mRNA expression of these genes, we collected the uterine horns of experimental mice for immunohistochemistry (IHC) and immunofluorescence (IF) staining. As shown in Figure S2, the expression pattern of Ihh, KLF9, MUC1, ERα, and PR in different groups of mice was consistent with the qRT-PCR results. Moreover, the increased expression of Ihh and KLF9 was observed both in the luminal epithelium and stroma in the uterus of injured mice (Figure S2A).

### 3.4. Ihh-KLF9-PR Signaling Pathway Mediated Regulation of ER/PR Balance in CRCs, Aberrant Activation in the Restoration of Injured Endometrium

**Figure 4:**
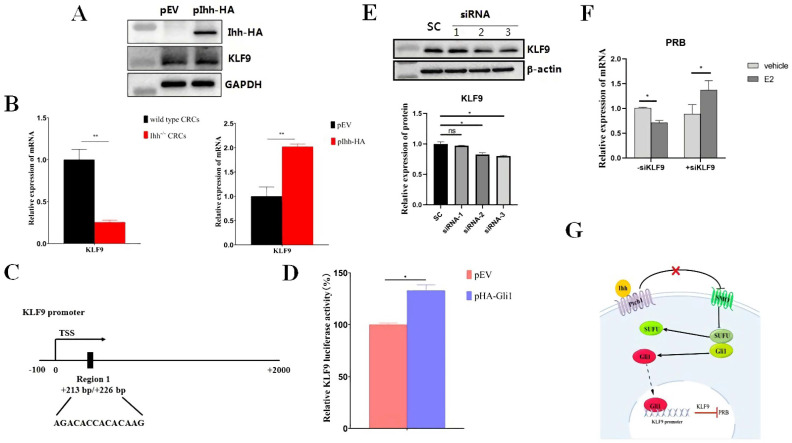
Ihh-KLF9-PR signaling pathway mediated regulation of ER/PR balance in CRCs, aberrant activation in the restoration of injured endometrium. (**A**) Over-expression of KLF9 in CRCs. CRCs were transfected with pIhh-HA and harvested after 24 h. The expression of Ihh and KLF9 was analyzed by Western blotting assay with primary antibodies against HA and KLF9. (**B**) KLF9 expression positively correlated with Ihh. The mRNA expression of KLF9 was detected using qRT-PCR in Ihh^−/−^CRCs or over-expressed Ihh CRCs. β-actin was an internal control, and the normalized expression level is displayed in a histogram (* *p* < 0.05, ** *p* < 0.01). The statistical analysis and plotting of the data were completed using GraphPad Prism 8.0. (**C**) The schematic diagram of the Gli1 binding region in the KLF9 promoter. TSS is designated as a transcription start site. (**D**) pHA-Gli1 activates KLF9 luciferase reporter. HEK293T cells were transfected with KLF9 luciferase reporter pKLF9-Luc and pPRL-TK, together with pHA-Gli1 or an empty vector (EV) for 36 h. The dual-luciferasee activity was then determined. Data were represented as relative firefly luciferase activity normalized by Renilla luciferase signals. (**E**) Knock-down KLF9 expression by siRNAs. The specific siRNAs targeting KLF9 were transfected into CRCs and the cell lysate was harvested after 72 h. The protein level of KLF9 was analyzed by Western blot assay and the relative amount of KLF9 was presented by densitometry quantitation (* *p* < 0.05, ns indicates no statistical significance). SC refers to scrambled siRNA. (**F**) mRNA expression of PRB in CRCs. PRB expression was detected by qRT-PCR after KLF9-siRNA was transfected into CRCs for 48 h and cells were treated with E2 for 24 h. (**G**) Diagram of Ihh-Gli1-KLF9-PR signaling pathway in the regulation of ER/PR balance in the endometrial epithelial cells. The diagram was created by Figdraw.

As our results have shown (Figure S1), Ihh is involved in the proliferation of endometrial epithelial cells. Transplantation of CRCs into IUA mice plays a dominant role during the restoration of uterine endometrium (Figure 3). Transplantation of endometrial CRCs in IUA mice could influence the re-epithelialization and microenvironment of the endometrium and in turn fibrosis formation. With the comparison of WT and Ihh^−/−^CRCs transplantation into IUA mice, we analyzed the expression of Ihh and KLF9 in the whole uterine tissue. Our results demonstrated that Ihh correlates with the expression of KLF9 (Figure 3). Next, we investigated whether Ihh signaling directly regulates KLF9 expression in CRCs. Ihh was over-expressed in CRCs (Figure 4A) or knockout by the CRISPR/cas9 method (Figure S1D). After the over-expression of Ihh, the mRNA and protein expression of KLF9 were both up-regulated in CRCs (Figure 4A,B) while the mRNA level of KLF9 was down-regulated in Ihh^−/−^CRCs (Figure 4B). These results demonstrated that KLF9 is positively correlated with Ihh in CRCs. As a ligand, Ihh binding to receptor Ptch1 relieves its repressive effect on the smoothened (SMO) protein an stabilizes and activates downstream transcription factor Gli1 (Figure 4G) [31]. The bioinformatic analysis using the JASPAR database (http://jaspar.genereg.net, accessed on 28 March 2022) predicts that Gli1 potentially binds to the KLF9 promoter (Figure 4C). Therefore, the KLF9 reporter pKLF9-Luc was constructed, and the luciferase reporter assay was performed. The results showed that the relative KLF9 luciferase activity was significantly enhanced by Gli1 over-expression compared to the empty vector (Figure 4D), suggesting the transcription of the KLF9 promoter is activated by the binding of Gli1. These results demonstrated that activation of Ihh signaling may regulate KLF9 expression in CRCs.

A previous study showed that KLF9 is a negative regulator and functions at the node of the PR and ER genomic pathways under estrogen control [32]. The specific siRNAs targeting KLF9 inhibited the expression of KLF9 in CRCs (Figure 4E). In the mouse uterus, PRB is the only functional PR in the luminal epithelium [33]. Therefore, we took the most efficient siRNA (#3 siRNA) to knock-down KLF9 and analyzed the expression of PRB with or without E2 treatment in CRCs. The results showed that the expression of PRB was down-regulated with E2 treatment when no KLF9-siRNA was with CRCs (Figure 4F). Knock-down of KLF9 by siRNA released the repression of KLF9 and significantly up-regulated the expression of PRB in CRCs (Figure 4F). These results demonstrated that KLF9 is a negative regulator of functional PR (PRB) under the physiological estrogen regulation in mouse endometrial epithelial cells. It is a reasonable speculation that Ihh signaling was aberrantly activated by injury and, in turn, up-regulated KLF9 and inhibited normal PRB function in IUA mice. The maintenance of endometrial homeostasis requires both ERα and PR and the two are in dynamic homeostasis [34]. Aberrant expression of PR may influence ER/PR balance in the endometrium, disturb the proliferation of endometrial epithelial cells, and lead to aggravated fibrosis in IUA mice.

Transplantation of endometrial CRCs in IUA mice could influence the re-epithelialization and microenvironment of the endometrium, and, in turn, fibrosis formation. Ihh is a regulator of progesterone signaling in the mouse uterus, mediating communication between the epithelium and stroma, suggesting the feedback regulation between Ihh and PR [4,30]. Our results demonstrated that aberrant activated Ihh-KLF9 signaling contributed to the inhibition of normal PR function in IUA mice, therefore affecting the restoration of injured endometrium.

### 3.5. Vismodegib Restores the Normal Microenvironment, Morphology, and Structure of Endometrium in IUA Mice

**Figure 5:**
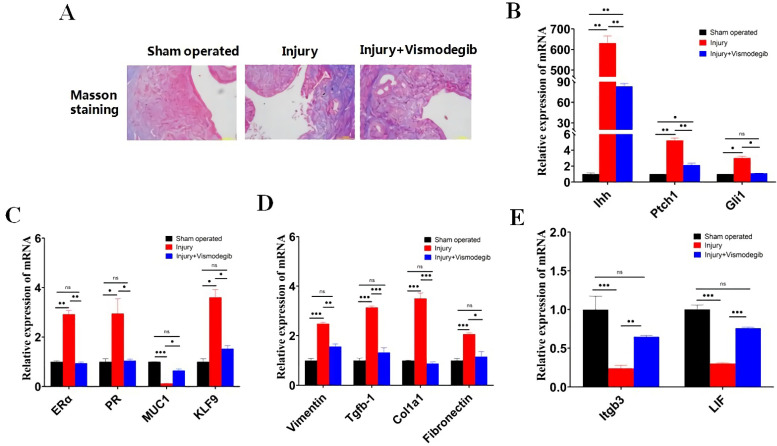
Vismodegib inhibits activation of the Ihh signaling pathway and fibrosis and recovers the receptivity of endometrium in IUA mice. (**A**) The endometrial morphology of the experimental groups of IUA mice treated with or without Vismodegib. Masson’s trichrome (MT) staining was performed to assess endometrial fibrosis. Scale bar, 100 μm. (**B**–**E**) RNA expression of Ihh signaling pathway molecules (**B**), key endometrial functional molecules (**C**), fibrosis markers (**D**), and endometrium receptivity markers (**E**) in the experimental groups of mice. Uterine endometrial tissues were collected, and qRT-PCR analysis was carried out. β-actin was the internal control and the normalized expression level is displayed in a histogram (* *p* < 0.05, ** *p* < 0.01, *** *p* < 0.001, ns indicates no statistical significance). The statistical analysis and plotting of the data were completed using GraphPad Prism 8.0.

Our previous results have shown that aberrant activation of Ihh signaling forced a negative microenvironment for IUA recovery. Therefore, we investigated whether the Hedgehog pathway inhibitor (Vismodegib) could rescue the inhibitory endometrium environment in mouse IUA mode. Six hours before mechanical damage to the endometrium, Vismodegib was administered to mice (Vismodegib treatment group) via gavage as described in Materials and Methods. The uterine tissues of the sham-operated group, injury group, and injury + Vismodegib group were collected after 7 days. Masson’s trichrome staining showed that the structure of the endometrium was recovered and fibrosis was reduced in the injury + Vismodegib group compared to that in the injury group (Figure 5A). The qPCR results showed that the mRNA expression of Ihh, Ptch1, and Gli1 was significantly down-regulated by Vismodegib treatment compared to that in injury mice, suggesting that Ihh signaling was significantly inhibited by Vismodegib (Figure 5B). Vismodegib also largely reduced the expression of KLF9 close to the control level (sham-operated group) (Figure 5C). As we expected, the expression of ERα and PR in Vismodegib-treated mice was down-regulated to the control level, suggesting that the aberrant repression of KLF9 was released and the normal microenvironment was restored by Vismodegib treatment (Figure 5C). The expression of MUC1 was increased to the control level in Vismodegib treated mice, suggesting the proliferation and restoration of endometrial epithelium by Vismodegib (Figure 5C). The immunohistochemistry (IHC) and immunofluorescence (IF) staining confirmed the expression pattern of Ihh, KLF9, MUC1, ERα, and PR after Vismodegib treatment and was consistent with the qRT-PCR results (Figure S3). In accordance with the restoration of endometrial epithelium by pretreatment of Vismodegib, the expression of fibrosis markers Vimentin, Tgfb-1, Col1a1, and Fibronect was all down-regulated to control level (Figure 5D). Compared to injury group mice, the expression of endometrial receptivity markers Itgb3 and LIF recovered close to the control level with Vismodegib treatment (Figure 5E). These results demonstrated that pretreatment with Vismodegib efficiently inhibited Ihh signaling, down-regulated KLF9, and restored the normal microenvironment, morphology, and structure of endometrium in IUA mice.

### 3.6. Vismodegib Treatment Improves Pregnancy Rate in IUA Mice

**Figure 6:**
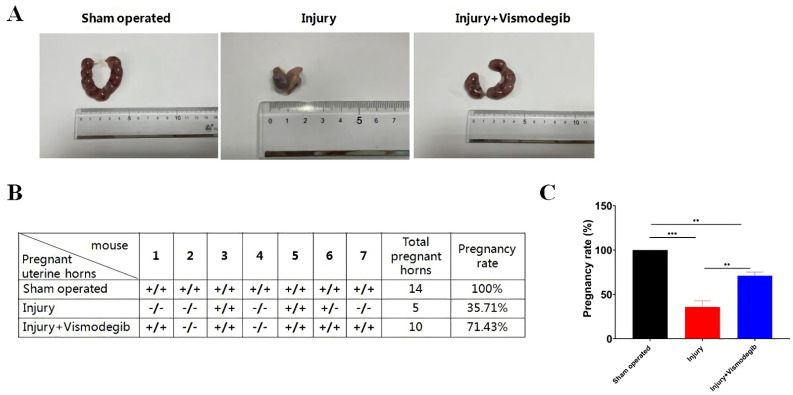
Vismodegib improves the pregnancy rate in IUA mice. (**A**) The representative appearance of embryo implantation in each group. Fourteen days (three estrous cycles) after injury, the female mice in three experimental groups were mated with male mice (1:1). The day was designated as gestation day (GD) 0 when a vaginal plug was observed. At GD 18, female mice were sacrificed for embryo implantation examination. (**B**) The number of pregnant uterine horns was counted and the pregnancy rate was calculated. (**C**) Statistical analysis of the pregnancy rate in each group by an ordinary one-way analysis of variance (ANOVA). Data were presented as the mean ± SD (*n* = 3). ** *p* < 0.01, *** *p* < 0.001.

To further evaluate the effect of Vismodegib on fertility, another batch of experimental mice was examined as a breeding study. The female mice were sacrificed and checked for embryo implantation at gestation day 18. In the sham-operated group, embryos were symmetrically distributed in uterine horns (Figure 6A). Compared to the sham-operated group, embryo malformation and fewer pregnant horns were observed in the injury group. Surprisingly, there were many more pregnant horns in the Vismodegib-treated group (Figure 6A,B). Mice from the sham-operated group were all pregnant (100%). Compared to the injury group, the pregnancy rate in the Vismodegib-treated group increased significantly from 35.71% to 71.43% (Figure 6B,C). These results demonstrated that treatment with Vismodegib could improve the pregnancy rate in IUA mice. 

## 4. Discussion

In recent years, the incidence of IUA has increased due to frequent intrauterine surgeries for clinical diagnosis and treatment [5]. Trauma to the endometrial basalis layer may wreak havoc on the regeneration of the endometrium and lead to IUA [35]. Current clinical treatment, hysteroscopic adhesiolysis, exhibits poor efficacy and prognosis for moderate to severe IUA patients. Therefore, alternative treatments for preventing recurrent adhesion after adhesiolysis are necessary.

Observation of the initial process of menstruation indicates the critical role of re-epithelialization of the endometrial surface in preventing fibrosis and scar formation [11,12]. Our previous study and the results of this study confirmed this hypothesis. Transplantation of mouse endometrial epithelial cells (as CRCs) in IUA mice significantly increased the expression of epithelial markers MUC1 (Figure 3E) and cytokeratin (CK)18 [14], and efficiently reduced the expression of fibrosis markers Vimentin, Tgfb-1, Col1a1, and Fibronectin (Figure 3D) [14].

Hedgehog (Hh) signaling regulates various development processes in vertebrates [31]. There are three Hh ligands, including Indian Hh (Ihh), Sonic Hh(Shh), and Desert Hh(Dhh) [36]. Ligand binding to patched receptors (Ptch1 and Ptch2) abolishes Ptch-inhibition on the smoothened (SMO) protein and activates transcriptional factors GLI (Gli1, Gli2, and Gli3) [37]. Ihh is expressed in the uterine epithelium under the control of progesterone [38,39] and is also a regulator of progesterone signaling in the mouse uterus, mediating the communication between the epithelium and stroma [30] and suggesting the feedback regulation between Ihh and PR [4]. The absence of Ihh in the mouse uterus results in decreased cell cycle progression and increased estrogen signaling [29]. It has been shown that down-regulated Ihh is linked to endometriosis [37,40]. However, little is known about the regulation and function of Ihh in IUA.

Krüppel-like factors (KLFs) belong to a 17-member family of transcriptional regulators expressed in reproductive tissues and function as co-regulators of steroid hormone actions [41]. A previous study has identified KLF9 as a direct PR-interacting protein [42,43] that functions as a transcriptional repressor at the node of the PR and ER genomic pathways to influence cell proliferation in the human endometrial adenocarcinoma cell line [32]. Our results found that the expression of Ihh and KLF9 were both up-regulated in the injury group mice. Injury-induced aberrant expression of Ihh signaling up-regulated KLF9 and thereafter inhibited the normal luminal expression of PRB in IUA mice. The normal function of ERα and PR are required to maintain endometrial homeostasis. The aberrant function of PR may influence the ER/PR balance in the endometrium, disturb the proliferation of endometrial epithelial cells, and lead to aggravated fibrosis in IUA mice. Although we observed the up-regulation of Ihh and KLF9 in the luminal epithelium and stroma in the uterus of the injury group mice, we could only explore the underlying mechanism by using long-term cultured CRCs. The cross-interaction of Ihh signaling and PR in the luminal epithelium and stroma warrants further investigation.

Considering the role of aberrant Ihh signaling in creating a negative microenvironment for IUA recovery, we investigated the role of the Hedgehog pathway inhibitor in the mouse IUA model. Vismodegib is a selective Hh pathway inhibitor approved by the US Food and Drug Administration (FDA) for the treatment of advanced basal cell carcinomas (BCCs) [44,45]. Our results demonstrated that pretreatment with Vismodegib efficiently inhibited Ihh signaling, down-regulated KLF9, restored the normal microenvironment, morphology, and structure of endometrium, and improved the pregnancy rate in IUA mice. Preclinical models and phase I clinical trials have shown the safety of this drug [22,44,45], suggesting Vismodegib to be a promising targeted drug in IUA therapy.

## Figures and Tables

**Table 1:** List of primer sets used in this study.

Gene		Sequences (5′-3′)	Amplicon Size (bp)
ERα	forward	CCCGCCTTCTACAGGTCTAAT	76
	reverse	CTTTCTCGTTACTGCTGGACAG	
PR	forward	CTCCGGGACCGAACAGAGT	128
	reverse	GCGGGGACAACAACCCTTT	
β-actin	forward	GTGACGTTGACATCCGTAAAGA	245
	reverse	GCCGGACTCATCGTACTCC	
Tgfb-1	forward	CTTCAATACGTCAGACATTCGGG	142
	reverse	GTAACGCCAGGAATTGTTGCTA	
Col1a1	forward	GCTCCTCTTAGGGGCCACT	91
	reverse	CCACGTCTCACCATTGGGG	
Vimentin	forward	CGGCTGCGAGAGAAATTGC	124
	reverse	CCACTTTCCGTTCAAGGTCAAG	
Fibronectin	forward	ATGTGGACCCCTCCTGATAGT	124
	reverse	GCCCAGTGATTTCAGCAAAGG	
Klf9	forward	GCCGCCTACATGGACTTCG	139
	reverse	GGTCACCGTGTTCCTTGGT	
Ihh	forward	GACGAGGAGAACACGGGTG	171
	reverse	GCGGCCCTCATAGTGTAAAGA	
Ptch1	forward	AAAGAACTGCGGCAAGTTTTTG	164
	reverse	CTTCTCCTATCTTCTGACGGGT	
Gli1	forward	CCAAGCCAACTTTATGTCAGGG	130
	reverse	AGCCCGCTTCTTTGTTAATTTGA	
MUC1	forward	GGCATTCGGGCTCCTTTCTT	132
	reverse	TGGAGTGGTAGTCGATGCTAAG	
Ki67	forward	ATCATTGACCGCTCCTTTAGGT	104
	reverse	GCTCGCCTTGATGGTTCCT	
Ccnd1	forward	GCGTACCCTGACACCAATCTC	183
	reverse	CTCCTCTTCGCACTTCTGCTC	
Itgb3	forward	GGCGTTGTTGTTGGAGAGTC	138
	reverse	CTTCAGGTTACATCGGGGTGA	
LIF	forward	ATTGTGCCCTTACTGCTGCTG	140
	reverse	GCCAGTTGATTCTTGATCTGGT	
PRB	forward	GGTCCCCCTTGCTTGCA	121
	reverse	CAGGACCGAGGAAAAAGCAG	

**Table 2:** List of primary antibodies used in this study.

Antibodies	Dilution	Application	Source	Catalog No.
Rabbit anti-Ihh	1:3000	Western blotting	abcam	ab39634
Rabbit anti-Ihh	1:100	Immunofluorescence	abcam	ab52919
Mouse anti-GAPDH	1:5000	Western blotting	Santa Cruz	sc-365062
Mouse anti-β-actin	1:5000	Western blotting	Santa Cruz	sc-47778
Mouse anti-ERα	1:200	Western blotting	Santa Cruz	sc-71064
Mouse anti-PR	1:200	Western blotting	Santa Cruz	sc-398898
Rabbit anti-KLF9	1:100	Immunofluorescence,	abcam	ab227920
	1:1000	Western blotting		
Rabbit anti-MUC1	1:100	Immunofluorescence	abcam	ab45167
Rabbit anti-Ki67	1:100	Immunofluorescence	abcam	ab16667
Rabbit anti-ERα	1:100	Immunohistochemistry	Proteintech	21244-1-AP
Rabbit anti-PR	1:100	Immunohistochemistry	CST	#8757
Mouse anti-HA	1:1000	Western blotting	Santa Cruz	sc-7392

## Data Availability

The data of this study are available upon reasonable request from the corresponding authors.

## Supplementary Materials

The following supporting information can be downloaded at: https://www.mdpi.com/article/10.3390/cells11244053/s1, Figure S1. Ihh gene targeting sites and validation by CRISPR/Cas9. Figure S2. In situ expression of key endometrial functional molecules in experimental groups of mice after transplantation of Ihh^−/−^CRCs or WT CRCs. Figure S3. In situ expression of key endometrial functional molecules in experimental groups of mice treated with or without Vismodegib.
