# Supplementary material for: CRC Therapy Identifies Indian Hedgehog Signaling in Mouse Endometrial Epithelial Cells and Inhibition of Ihh-KLF9 as a Novel Strategy for Treating IUA
Source: Cells. 2022 Dec 15;11(24):4053. doi: 10.3390/cells11244053 (PMC9776583; doi:10.3390/cells11244053)
Supplement: Supplementary file 1 [file cells-11-04053-s001.zip › cells-2031100-supplementary.pdf]

CRC therapy identifies Indian hedgehog signaling in mouse endometrial epithelial cells and inhibition of Ihh-KLF9 as a novel strategy for treating IUA

Xinhao Zhou[1,2#], Yiyi Kang[1#], Yun tzu Chang[3#], Siyu Xia[1], Ming Wu[1], Jun Liu[1], Dirong Dong[3], Wei Zhang[3], Hong Chen[3]*, Hui Li[1]*

Supplementary Materials

A

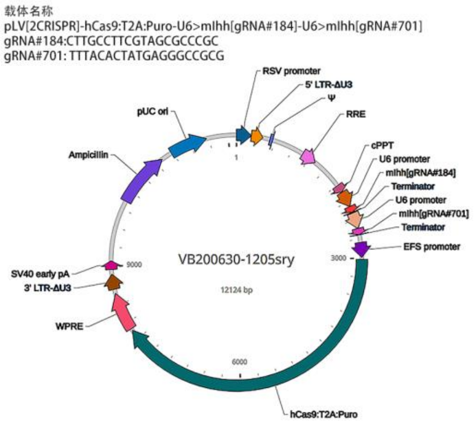

B

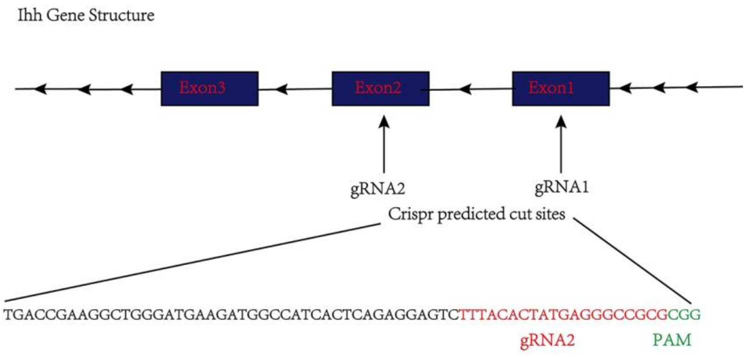

C

	targetted Ihh sites
Reference Sequence (wild type)	TTTACACTATGAGGGCCGCGCGG
Clone 1 (Insertion)	TTTACACTATGAGGGCCCGCGCGG
Clone 2 (Insertion and Mutation)	TTTACACTATGAGGGCCCGCACGG
Clone 3 (deletion)	TTTACACTATGAGGG_CGCGCGG

D

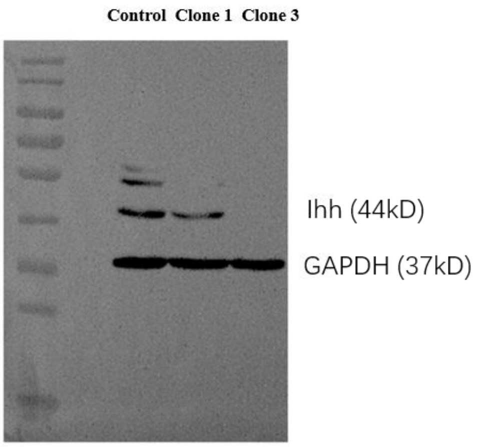

E

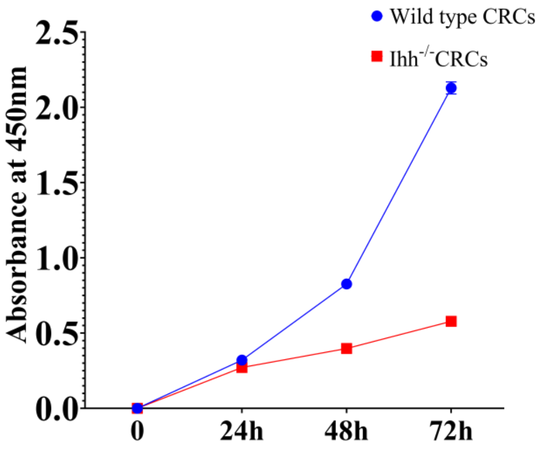

Figure S1. Ihh gene targeting sites and validation by CRISPR/Cas9. (A)Schematic diagram of CRISPR/Cas9 lenti-virus vector structure (mammalian gRNA and Cas9 co-expression vector). (B)Ihh gene cutting sites by CRISPR/Cas9. The two gRNAs were designed on exon 1 and exon 2 of Ihh gene respectively. (C) Sequencing results of clones edited by CRISPR/Cas9. Three clones

were selected and sequenced. (D) Validation of Ihh protein expression in subclones. Western blotting analysis was used to detect the Ihh proteins in clone 1 and 3. The empty vector of lentivirus was used as the control. GAPDH is an internal control. (G) Cell proliferation rate of $Ihh^{-/-}$and wildtype (WT) CRCs cells. Cells were seeded at a density of 5×10^3cells/well on 24 well plates. The cell proliferation assay was performed using a Cell Counting Kit 8. The absorbance was measured at 450 nm with a microplate reader. The experiment was repeated in triplicate.

A

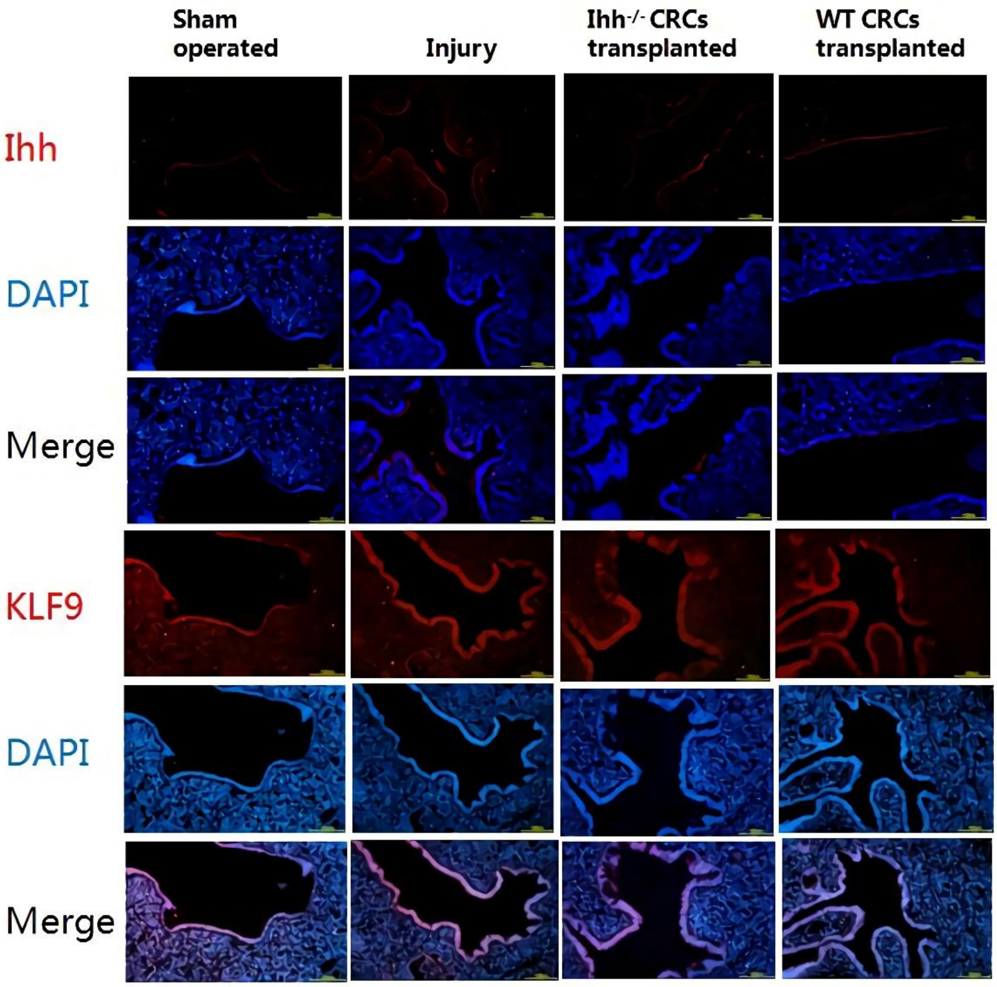

B

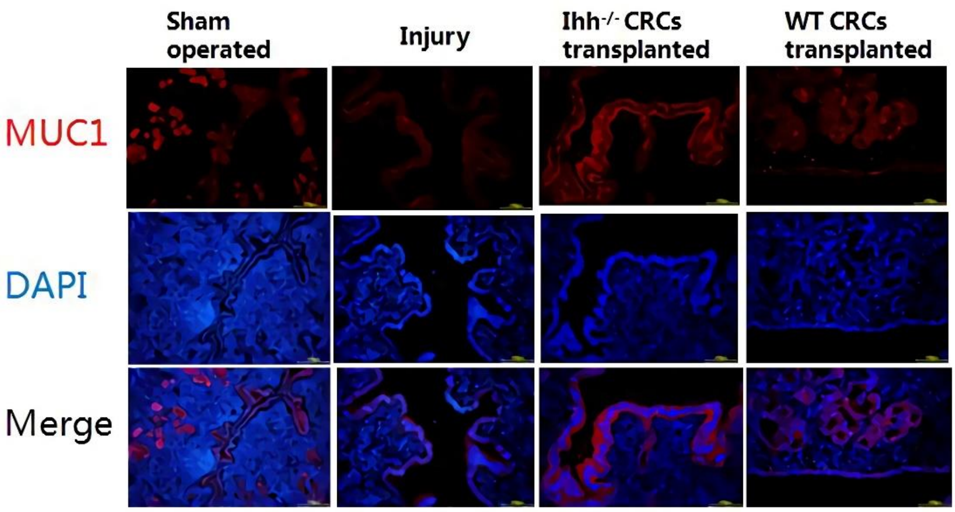

C

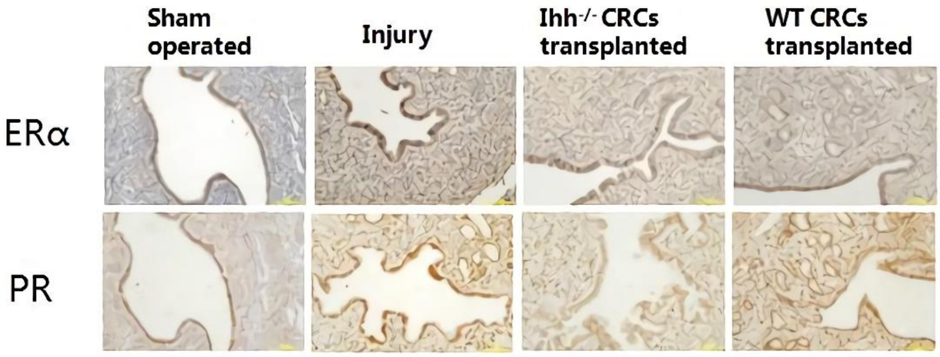

Figure S2. In situ expression of key endometrial functional molecules in experimental groups of mice after transplantation of Ihh$^{-/-}$ CRCs or WT CRCs. The uterine horns of experimental groups were collected at 7 days after surgery/cells transplantation. The expression of Ihh and KLF9 (A), MUC1 (B) was detected by immunofluoresence (IF) staining. Nuclei were stained blue with DAPI. The expression of ERα and PR was detected by Immunohistochemical staining (C). Brown denotes ERα and PR-positive cells. Scale bars represent: 50 μm in A and B; 100 μm in C.

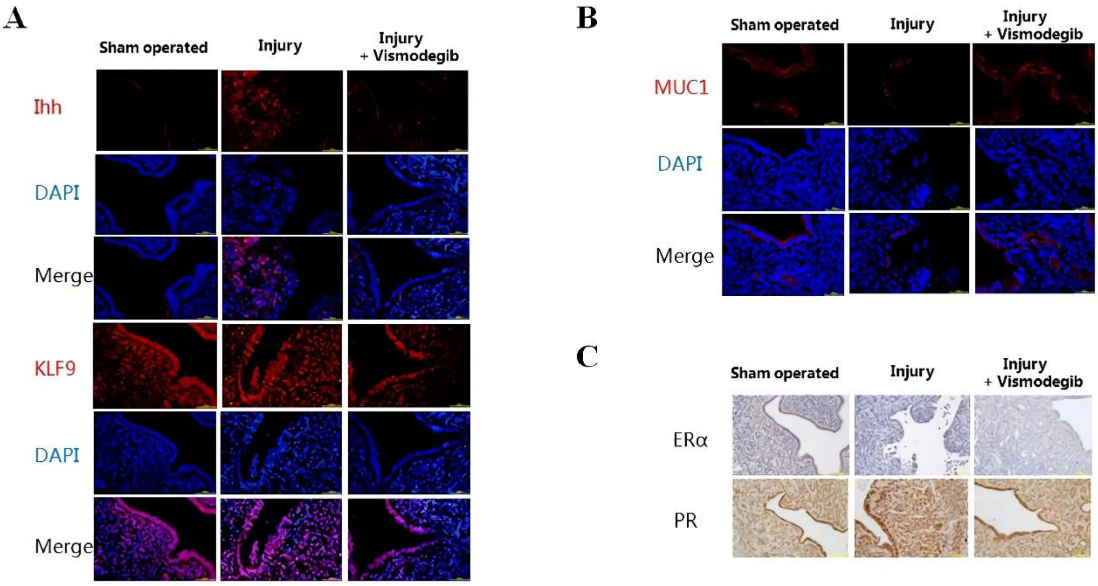

Figure S3. In situ expression of key endometrial functional molecules in experimental groups of mice treated with or without Vismodegib. The uterine horns of each group were collected at 7 days after surgery. The expression of Ihh and KLF9 (A), MUC1 (B) were detected by IF staining. Nuclei were stained blue with DAPI. The expression of ERα and PR was detected by Immunohistochemical staining (C). Brown denotes ERα and PR-positive cells. Scale bars represent: 50 μm in A and B; 100 μm in C.
